# An Automated Broncho-Arterial (BA) Pair Segmentation Process and Assessment of BA Ratios in Children with Bronchiectasis Using Lung HRCT Scans: A Pilot Study

**DOI:** 10.3390/biomedicines11071874

**Published:** 2023-06-30

**Authors:** Sami Azam, Sidratul Montaha, A. K. M. Rakibul Haque Rafid, Asif Karim, Mirjam Jonkman, Friso De Boer, Gabrielle McCallum, Ian Brent Masters, Anne Chang

**Affiliations:** 1 Faculty of Science and Technology, Charles Darwin University, Casuarina, NT 0909, Australia; sidratul15-11685@diu.edu.bd (S.M.); rakibul15-11463@diu.edu.bd (A.K.M.R.H.R.); asif.karim@cdu.edu.au (A.K.); mirjam.jonkman@cdu.edu.au (M.J.); friso.deboer@cdu.edu.au (F.D.B.); 2 Child Health Division, Menzies School of Health Research, Darwin, NT 0811, Australia; gabrielle.mccallum@menzies.edu.au (G.M.); anne.chang@menzies.edu.au (A.C.); 3 Australian Centre for Health Services Innovation, Queensland University of Technology, Brisbane, QLD 4059, Australia; ianbrentmasters@gmail.com; 4 Department of Respiratory and Sleep Medicine, Queensland Children’s Hospital, South Brisbane, QLD 4101, Australia

**Keywords:** broncho-arterial pair, broncho-arterial ratio, image processing, HRCT scans

## Abstract

Bronchiectasis in children can progress to a severe lung condition if not diagnosed and treated early. The radiological diagnostic criteria for the diagnosis of bronchiectasis is an increased broncho-arterial (BA) ratio. From high-resolution computed tomography (HRCT) scans, the BA pairs must be detected first to derive the BA ratio. This study aims to identify potential BA pairs from HRCT scans of children undertaken to evaluate suppurative lung disease through an automated approach. After segmenting the lung regions, the HRCT scans are cleaned using a histogram analysis-based approach followed by a potential arteries identification process comprising four conditions based on imaging features. Potential arteries and their connected components are extracted, and potential bronchi are identified. Finally, the coordinates of potential arteries and potential bronchi are matched as the last step of BA pairs extraction. A total of 8–50 BA pairs are detected for each patient. Additionally, the area and several diameters of the bronchi and arteries are measured, and BA ratios based on these are calculated. Through this approach, the BA pairs of a CT scan datasets are detected and utilizing a deep learning model, a high classification test accuracy of 98.53% is achieved, validating the robustness of the proposed BA detection approach. The results show that visible BA pairs can be identified and segmented automatically, and the BA ratio calculated may help diagnose bronchiectasis with less effort and time.

## 1. Introduction

Bronchiectasis is a serious lung condition caused by bacterial inflammation and can result in a low life expectancy. It can be described by irreparable bronchial dilatation [1]. Bronchiectasis is common in indigenous children in Australia, with signs early in life [2]. Bronchiectasis in children and adults has similarities but also considerable differences. In cystic fibrosis (CF) infants, bronchiectasis has been detected at ten weeks of age [3,4]. In 3–5 years old patients with CF, 50–70% are affected by bronchiectasis [5]. Bronchiectasis, unrelated to CF, is also increasing in children and adults [6]. To prevent further progression of bronchiectasis, early detection is crucial [7,8,9]. At present, the gold standard in diagnosing bronchiectasis is high-resolution computed tomography (HRCT), where an increased broncho-arterial (BA) ratio is regarded as one of the key markers [10,11]. However, the BA ratio of children and adults may differ as lung morphology and structure alter with age [9]. The exact BA ratio, which indicates bronchiectasis is a topic of debate. The BA ratio can be measured from CT scans by expert radiologists using electronic calipers [9]. Naseri et al. [12] employed image preprocessing, threshold-based and model-based techniques to distinguish airways and lung vessels using HRCT images. Prasad et al. [13] applied multi-view and active learning approaches in detecting bronchial dilatation using lung HRCT images. Gao et al. [14] segmented bronchi, arteries and pulmonary veins from CT images using Amira 4.1 software (Thermo Fisher Scientific; Waltham, MA, USA) and marked each component differently. To detect valid and invalid airway walls, Schmidt et al. [15] introduced image preprocessing methods for CT scans based on local intensity levels. For the segmentation of airway trees, Meng et al. [16] applied image preprocessing and a support vector machine algorithm on lung CT scan images. Prasad and Sowmya [17] extracted the lung regions from CT scans, applying image preprocessing methods. A region-growing algorithm was applied to detect bronchi and the broncho-vascular pairs through co-training and an active learning approach. Perez et al. [18] conducted lung segmentation from a CT scan using a Hessian eigenvalue analysis algorithm. Airways and vessels were extracted using image preprocessing methods. Zrimec and Busayarat [19] detected bronchi and arteries employing the local intensity gradient method, a region-growing algorithm with leaking correction and a rule-based classification approach using CT scan images. Busayarat and Zrimec [20] detected bronchi and arteries using image preprocessing algorithms to evaluate bronchial dilatation. Though the bronchial dilatation cutoff is defined as a BA ratio larger than 1 [21], this ratio is smaller in younger people. Several bronchiectasis studies have reported a lower BA ratio in young adults than in older ones [22]. Park et al. [23] observed an average BA ratio 0.65 while studying healthy subjects. A BA ratio of 0.62 ± 0.02 was reported by Berend et al. [24] for the normal lungs of four subjects. Matsuoka et al. [22] found that for the age group of 20–40, the mean BA ratio is 0.609 ± 0.05, whereas, for patients over 65, the mean BA ratio is 0.782 ± 0.08. Kapur et al. [9] found BA ratios in the range of 0.437–0.739 for children. Therefore, determining the BA ratio in children has become a key research interest. However, detecting BA pairs manually is time-consuming and often tedious and error-prone [18]. An automated system to detect BA pairs from HRCT scans might be a suitable approach [12,13]. This research, therefore, aims to detect BA pairs from CT scan images to diagnose bronchiectasis in children through an automated approach.

The significant contributions of this research are as follows:

- To identify BA pairs, lung segmentation and image cleaning are conducted using several image preprocessing and custom-developed algorithms.
- Potential bronchi and arteries are identified by applying several algorithms based on their characteristics, and the BA pairs are extracted by matching the coordinates of potential bronchi and arteries.
- The BA ratio of the detected BA pares is determined in an automated approach, and the results are evaluated and validated based on human assessment and deep learning-based techniques.

## 2. Methods

### 2.1. Study Design

The recruitment of children was done prospectively at Royal Darwin Hospital, Northern Territory, Australia, as a part of a study concerning bronchoscopy in children having chronic wet cough. The children undergoing bronchoscopy were registered successively. For the present research, the HRCT scans of the children are used for further investigation with a computerized approach. This study was approved by the local Human Research Ethics Committee of the Northern Territory Department of Health and Menzies School of Health (Identifier: HREC 07/63) on 22 April 2022.

### 2.2. Participants

The eligibility of the children is defined after the primary testing of their clinician. They were clinically stable during the investigation and referred to the Royal Darwin Hospital for further analysis. Children formerly diagnosed with bronchiectasis or CF were omitted from the analysis. HRCT scans of the patients are taken under general anesthesia. Since HRCT scans acquired during acute infection do not reveal the nonacute state, no examination is commenced during an acute respiratory infection. During the enrolment, children’s medical information, such as demographic data, medical history, and clinical data, were acquired from their primary caregiver and medical records, including prior respiratory hospitalizations and medication usage.

### 2.3. Sample Collection

Both manual and automated diagnostic investigations of HRCT are greatly influenced by the parameters used. Knowledge related to anatomy, pathophysiology, and the underlying physics and engineering concepts of CT is required for the optimal acquisition of HRCT scans. This study uses a dataset consisting of HRCT scans of nine children with ages ranging from 1 year two months to 8 years four months, with symptoms indicative of bronchiectasis. All the scans are taken with the Philips Ingenuity Core CT scanner and the Toshiba Aquilion CT scanner at Royal Darwin Hospital. A high-resolution 64 slice 137 Multi-Detector CT (MDCT) obtained near-isotropic data throughout the thorax in one particular breath-hold. This allows the acquirement of volumetric single-breath-hold datasets with spaced, adjoining, and/or corresponding HRCT scans. The multi-planar (axial, coronal, sagittal) thin-section HRCT scans can be reconstructed with MDCT. For each subject, the HRCT scans used in this research have approximately nine forms of reconstructed images from diverse planes. No contrast enhancement was used, and the CT scans were generated using a normal reconstruction kernel in Digital Imaging and Communications in Medicine (DICOM) format. The HRCT scans consist of approximately 20–700 slices. The proposed methods of this study are applied to the axial plane of the images, with a slice thickness and interval of 0.67 mm. Table 1. provides the dataset details, including data acquisition information, technical parameters details and patient details.

**Figure 1:**
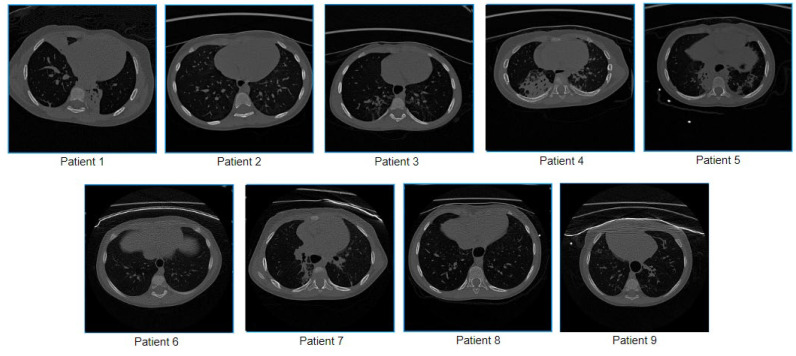
Sample images of nine children utilized in this study.

Sample images of the nine children are shown in Figure 1.

To suit our computer vision-based approach, the scans are converted to 512 × 512 greyscale images. Patient data such as age, name, and date of birth have been removed from the images.

Another public lung CT scan dataset [25] validates our proposed computerized approach. This dataset includes 17,104 scans, of which 6983 are non-COVID subjects, 7593 are from COVID patients, and the remaining 2618 are of community-acquired pneumonia (CAP) patients. These scans are obtained from a total of 3688 subjects. All the CT scans are in Portable Network Graphics (PNG) format and of 512 × 512 pixels size.

**Figure 2:**
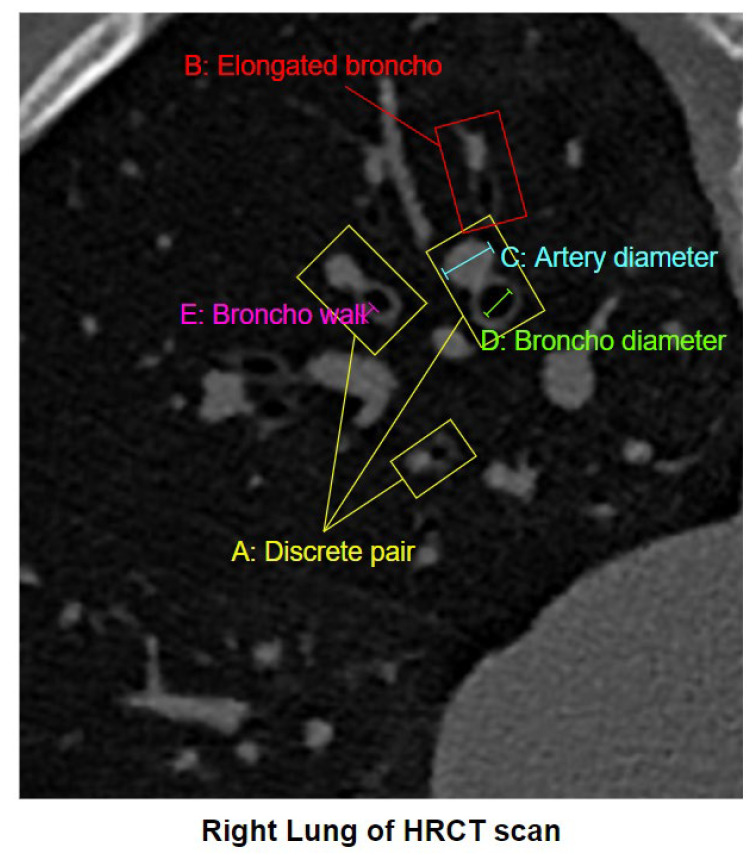
Properties relevant for diagnosing are illustrated in an original HRCT scan of the right lung.

Figure 2 illustrates some properties relevant to diagnosing bronchiectasis.

In Figure 2, A denotes a discrete BA pairs, B represents an elongated BA pair, C shows the diameter of an artery, D indicates the diameter of a bronchus, and E refers to the bronchial wall. In diagnosing bronchiectasis, one of the most important biomarkers is bronchial dilatation, as determined by the BA ratio from discrete and “signet-ring” shaped BA pairs [26]. A discrete BA pair is a single broncho adjacent to a single artery. Bronchial dilatation is measured by dividing the bronchial inner diameter by the arterial diameter, illustrated in Figure 2 by D and C, respectively [20]. In elongated BA pairs, the broncho-artery pairis found to be elongated instead of a nearly circular shape. Clinically, these elongated pairs are often not taken into account for the diagnosis of bronchiectasis. Bronchial wall thickening is another major aspect of bronchiectasis [27]. To find the presence and degree of wall thickening, the thickness of the bronchial wall needs to be determined.

## 3. Test Methods

**Figure 3:**
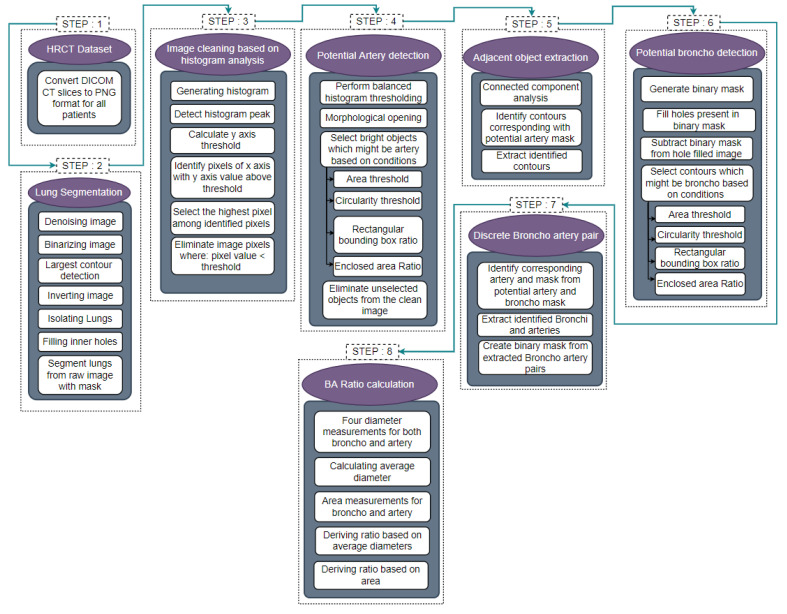
Process of the proposed BA pair segmentation approach.

This research aims to segment the BA pairs using HRCT images of the lungs. The BA pairs are detected and segmented using different image preprocessing algorithms. The major steps are image cleaning, potential artery detection, potential bronchus detection, BA pair identification and BA ratio measurement. Figure 3 illustrates the methodology used to extract BA pairs.

Interpreting CT scan data to detect BA pairs is challenging due to noise, artifacts, cardiac motion while scanning, partial volume effects, broken bronchial walls and differences in contrast levels in different images [13]. Initially, the HRCT images in DICOM format are converted to 2D slices in PNG format. Lung segmentation for all slices is carried out in six steps: denoising the image, binarizing the image, largest contour detection, inverting the image, hole-filling, and extracting the lung regions. After segmenting the lungs, the images are cleaned using a histogram analysis-based approach using a dynamic threshold. To identify BA pairs, potential arteries and bronchi need to be detected first. For detection of the arteries, balanced histogram thresholding and morphological opening are applied to extract the bright regions of the image. Identifying these regions as arteries are based on object area, circularity, rectangular boundary box ratio and enclosed circle area ratio. Threshold values for these properties are specified. Objects which are close to circular shapes are considered potential arteries. Objects adjacent to these potential arteries are extracted using connected component analysis and coordinate matching methods. Images containing potential arteries with adjacent objects are used to find potential bronchi. The images are converted to a binary format, and all the holes present in the images are filled using a hole-filling algorithm. The binary image is then subtracted from the hole-filled image, resulting in an output image containing potential bronchi.

Similar to the detection of potential arteries, four conditions are applied to extract circular-shaped holes, which could be potential bronchi. The discrete BA pairs can then be identified by matching the coordinates of potential arteries with potential bronchi. For the performance evaluation of this BA pair detection approach, a public CT scan dataset is used, comprising three classes (COVID, Non-COVID, CAP). Utilizing the proposed approach, BA pairs of the public CT scan dataset are detected. These modified CT scans are used for training a deep learning model to evaluate the effectiveness of the proposed BA pair detection approach. Finally, the BA ratio is calculated based on the diameter and area of the bronchi and arteries. Further details of the process are explained in the following sub-sections.

### 3.1. Lung Segmentation

**Figure 4:**
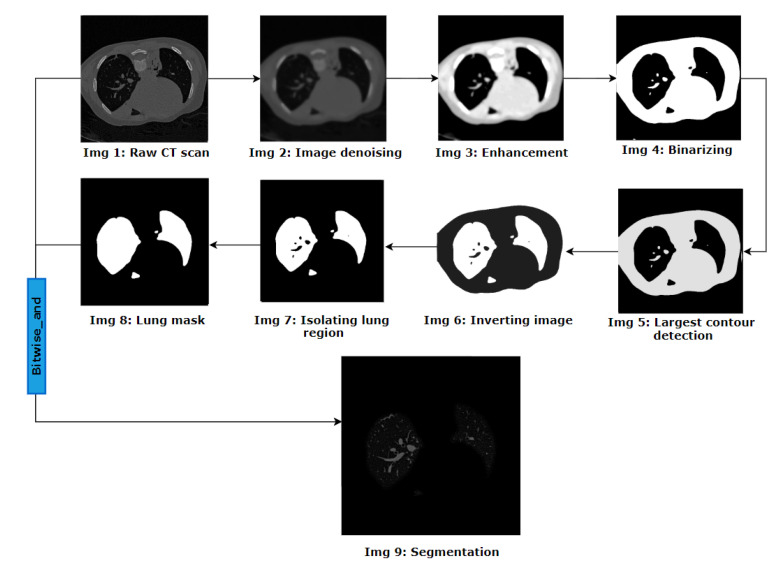
Lung segmentation process.

Detection of BA pairs from HRCT scans is quite challenging as these often have complex structures, broken regions, low contrast, and different intensity levels for different images. The images should therefore be made as clean as possible. In a CT scan, relevant information for bronchiectasis, such as BA pairs, is located in the lung regions. The outer regions of the images are not helpful for the detection and assessment of BA pairs. Hence, the two lungs are extracted first from the CT scans, employing different image preprocessing techniques. Figure 4 illustrates the methodology used to remove the lung regions from the CT scans.

This automated approach applies a suite of algorithms to all the 2D axial slices of the CT scans. The noise of the original images is removed using the total variation denoising technique, which preserves the edge information (Img 2 of Figure 4). To define the lung regions better, the conditional alpha beta correction method is applied to dynamically adjust the overall brightness and contrast of the denoised image based on the mean pixel value for each image. The images are then converted to binary format using the Otsu thresholding algorithm. In this way, the pixel value of most of the lung regions becomes 0. However, some remaining artifacts in the images can be eliminated by means of the largest contour detection technique. The resultant image is inverted, meaning the lung areas become white, surrounded by black regions. Employing the flood fill algorithm to the inverted image, the pixel value of the regions outside the lungs is turned to 0. Objects such as bronchi, arteries and veins are still visible inside the lungs. To acquire a binary mask containing only the lung regions, these are eliminated using the morphological reconstruction method. The binary mask thus obtained is then merged with the original image to segment the lung regions.

### 3.2. Image Cleaning Based on Histogram Analysis

**Figure 5:**
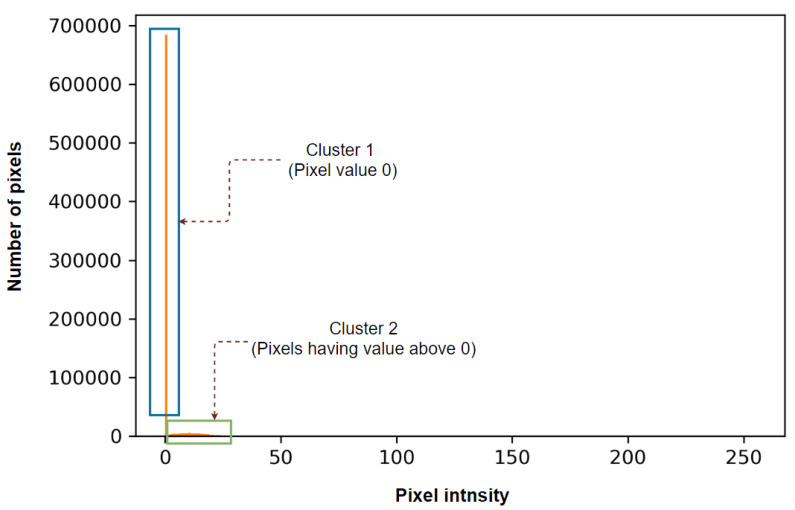
Histogram plot after segmenting the lung portions.

A histogram plot is derived from the lung-segmented image, and a threshold pixel value is determined for every image based on the histogram outline. This threshold eliminates tiny noises and unwanted regions of the image. First, the segmented image is converted into a 2D array, and the histogram plot is generated, as shown in Figure 5.

**Figure 6:**
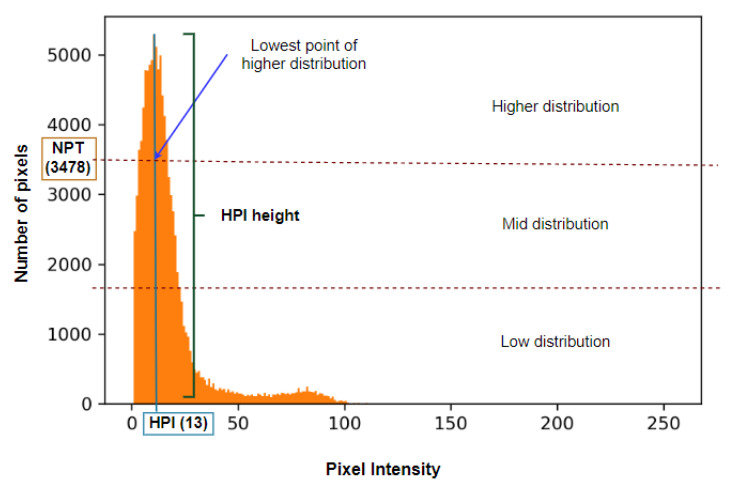
Histogram plot after removing all the pixel values of 0 and determining the number of pixel thresholds (NPT).

For better understanding, the histogram plot of Figure 5 is split into two clusters. Cluster 1 contains only the pixels with an intensity of 0, and Cluster 2 includes all others. As the pixel value 0 (cluster 1) is the most common, different intensity levels (cluster 2) can barely be seen in the plot. This is caused by the fact that in the segmented lung images, the background (pixel value 0) covers most of the image area. For a better visualization of cluster 2, all the 0 values are removed, and the histogram plot is generated again. Figure 6 shows the histogram after removing all the pixel values of 0.

**Figure 7:**
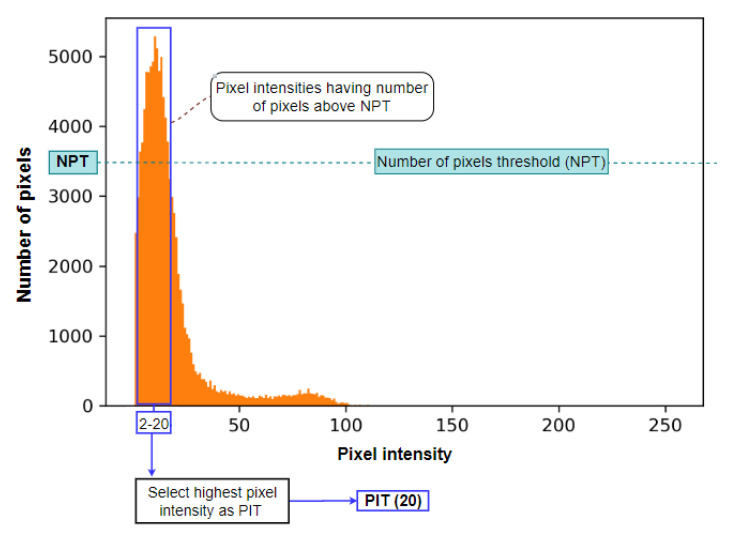
Process of determining the pixel intensity threshold (PIT) from the number of pixel thresholds (NPT).

It can be observed from Figure 6 that after discarding the pixel values of 0, pixels of other intensity levels are visible. This approach aims to discard as many unwanted pixels as possible without affecting the BA region. To achieve this, two threshold values are derived from the histogram: the number of pixel thresholds (NPT) and the pixel intensity threshold (PIT). The PIT is based on the value of the NPT. To determine NPT, the highest peak intensity (HPI) of the histogram distribution is considered. Utilizing the HPI height, the histogram plot is horizontally divided into three distributions, a high distribution, a middle distribution, and a low distribution. The value of the NPT is the lower limit of the increased distribution of the histogram. For this particular CT scan histogram (Figure 6), the HPI value is 13. After dividing the histogram into three distributions, the lower limit of the high distribution, the NPT, is found to be 3478. If we observe the original image (Img 8 of Figure 4), it can be seen that there is a large number of tiny noises with comparatively low pixel intensities values. The method of deriving the PIT using NPT is visualized in Figure 7.

As can be seen in Figure 7, the NPT determines the PIT. The pixel intensities with several pixels larger or equal to the NPT are within the blue box. The highest intensity within this range is the PIT. For the CT scan histogram example of Figure 7, it can be seen that the pixels in the blue box have intensity levels in the range of 2 to 20. The highest intensity, 20, is selected as the threshold for this particular CT scan, and all the pixels with intensities below 20 are removed. The histogram could be divided into more distribution levels (Figure 6), resulting in different threshold values. After experimentation with different numbers, it was found that dividing the histogram into three distributions resulted in the best output. The pseudo-code of Algorithm 1 describes all the steps of the histogram-based cleaning process.
**Algorithm 1:** Pseudo-Code of Histogram Analysis-Based Image Cleaning.**START**Read input image, im(x,y)Convert im(x,y) into 2D array, a(x,y)Derive the first histogram, FH, from a(x,y)Remove all the 0 values from a(x,y)Derive the second histogram, SH, from a(x,y)Determine the highest peak intensity value, HPIDerive height of HPICluster SH based on HPI height along y axis into three distributions, high distribution (HD), mid distribution (MD) and low distribution (LD)Determine the number of pixel threshold, NPT from the low point of HDDetermine PIT from NPTFor pixel intensity 0–255 in SH along the x-axis:  **If** a number of pixels along the y-axis ≥ NPT:  Keep pixel intensity in the PIT thresholdRemove pixels from im(x,y) using the threshold, PIT where im(x,y) ≤ PIT**END**

**Figure 8:**
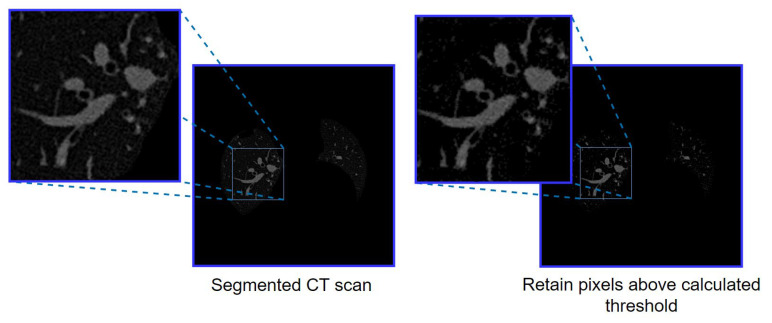
The output of image cleaning is based on histogram analysis.

Figure 8 shows the output (with a zoomed portion of the left lung) after removing all the pixels below the threshold level.

Figure 8 shows that after removing all pixels under the threshold level, the majority of tiny noises are removed. Analysing the histograms and determining the threshold values is a dynamic process to remove the noise from all images.

### 3.3. Potential Artery Detection

Detecting potential arteries is done in three steps: balanced histogram thresholding, morphological opening, and condition-based potential artery detection.

#### 3.3.1. Balanced Histogram Thresholding and Morphological Opening

**Figure 9:**
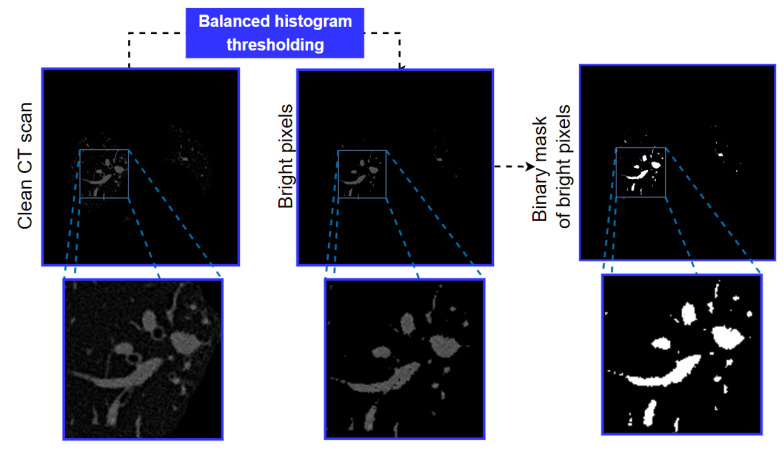
Output after applying the balanced histogram thresholding method.

The arteries are some of the brightest regions of the cleaned image (Figure 8). The bright regions of the cleaned image are therefore extracted first, utilizing balanced histogram thresholding. Balanced histogram thresholding is an automated thresholding algorithm which balances the pixel distribution of the image histogram and automatically determines a threshold. It can extract bright regions of the image without losing necessary details. After applying the algorithm, the image is turned into a binary format. Figure 9 shows the binary image after applying the balanced histogram thresholding method to the input image.

After completing the process, it is found that only the brightest areas, which may be potential arteries, are left in the image. To eliminate bright areas which can be noisy, we then apply morphological opening with a kernel size of 3 × 3 [28].

#### 3.3.2. Condition-Based Potential Artery Detection

Condition-based techniques are used to identify potential arteries. The connected components of the images are detected first using connected component analysis techniques. To detect potential arteries, the connected components of the resultant images are sorted based on four conditions.

- Condition-1: Object area within threshold limits
- Condition-2: Object circularity above a threshold value
- Condition-3: Height to the width of the ratio of rectangular bounding box above a threshold value
- Condition-4: Ratio of the area to the area of the enclosed circle above a threshold value.

For the object area-based condition, the area of each connected component in the image is derived. Objects having an area <10 or >300 could potentially be arteries. Objects with a pixel size <10 are expected to be too small objects with a pixel size >300 are too large to be bronchial arteries. Therefore, the thresholds to retain the potential arteries are set to 10 and 300. Algorithm 2 describes the process of retaining objects based on their area.
**Algorithm 2:** Object Area-Based Potential Artery Extraction Process.1. **START** 2. Read input image im(x,y) 3. Find each connected component, C, from im(x,y) 4.    **FOR** C in im(x,y): 5.      Derive the area, A of C 6.    **IF** A > 10 | A < 300 7.      Keep C in im(x,y) 8.    **ELSE** 9. Discard C from im(x,y) 10.    **END IF** 11. **END FOR** 12. **END**

For the object circularity-based condition, the circularity of each connected component in the image is derived. The equation to calculate the circularity of an object is as follows [29]:

(1) $$Circularity=4\pi *\frac{\left[\;\mathrm{Area}\;\right]}{{\left[\;\mathrm{Perimeter}\;\right]}^{2}}$$

For a circle, the circularity is equal to 1. For non-circular objects, the value is less than 1. In this case, connected components with a circularity >0.3 are retained. Algorithm 3 explains the process of retaining objects based on circularity.
**Algorithm 3:** Object Circularity-Based Potential Artery Extraction Process. 1. **START** 2. Read input image im(x,y) 3. Find each connected component, C, from im(x,y) 4. **FOR** C in im(x,y): 5.    Derive the circularity, Cr of C 6.    **IF** Cr > 0.3 7.      Keep C in im(x,y) 8.    **ELSE**
9.      Discard C from im(x,y) 10.   **END IF** 11. **END FOR** 12. **END**

For the rectangular boundary box-based condition, a rectangular-shaped bounding box surrounding each object of the image is drawn using the four coordinates of an object. The coordinates are determined so that the enclosed bounding box has the smallest possible area while covering the object completely. The rounder an object is, the closer the bounding box of that object will be to a square. This means that the height-width ratio is close to 1. In this case, the longest side of the bounding box is defined as the height and the shortest side is defined as the width of the bounding box. The ratio of the two is computed using the following equation:

(2) $$BBRatio=\frac{W}{H}$$

here, *BBRatio* denotes the bounding box ratio, *W* denotes the width, and *H* denotes the height. To extract potential arteries, the threshold is set to 0.4. Algorithm 4 explains retaining objects based on the rectangular bounding box.
**Algorithm 4:** Rectangular Boundary Box-Based Potential Artery Extraction Process.1. **START** 2. Read input image im(x,y) 3. Find each connected component, C from im(x,y) 4. **FOR** C in im(x,y): 5.    Determine four co-ordinates, W, X, Y, Z 6.    Draw a rectangular bounding box, RB through W, X, Y, Z 7.    Denote H = height of the bounding box 8.    Denote W = width of the bounding box 9. **IF** H > W: 10. Ratio, R = W / H 11. **ELSE** 12. R = H / W 13. **IF** R > 0.4 14. Keep C in im(x,y) 15. **ELSE**
16.      Discard C from im(x,y) 17.    **END IF** 18. **END FOR** 19. **END**

For the enclosed circle-based condition, a circle is drawn surrounding each object. The area of the enclosed circle and the object is compared using the following equation:

(3) $$ECRatio=\frac{O}{E}$$

where, *ECRatio* denotes the ratio, *O* denotes the object area, and *E* denotes the enclosed circle area. In this regard, the larger the relative difference between the enclosed circle area and the object area, the less circular object is. For a perfect circle, the ratio is 1. To find potential arteries, the ratio threshold is set to 0.4. Algorithm 5 explains the process of retaining objects based on this condition.
**Algorithm 5:** Enclosed Circle-Based Potential Artery Extraction Process.1. **START** 2. Read input image im(x,y) 3. Find each connected component, C from im(x,y) 4. **FOR** C in im(x,y): 5.   Determine four co-ordinates, W, X, Y, Z 6.    Draw an enclosed circle, Cr through W, X, Y, Z 7.    Derive area of C = AC 8.    Denote area of Cr = ACr 9.    Ratio, R = AC / ACr 10.    **IF** R > 0.4 11.     Keep C in im(x,y) 12.   **ELSE**
13.      Discard C from im(x,y) 14.    **END IF** 15. **END FOR** 16. **END**

**Figure 10:**
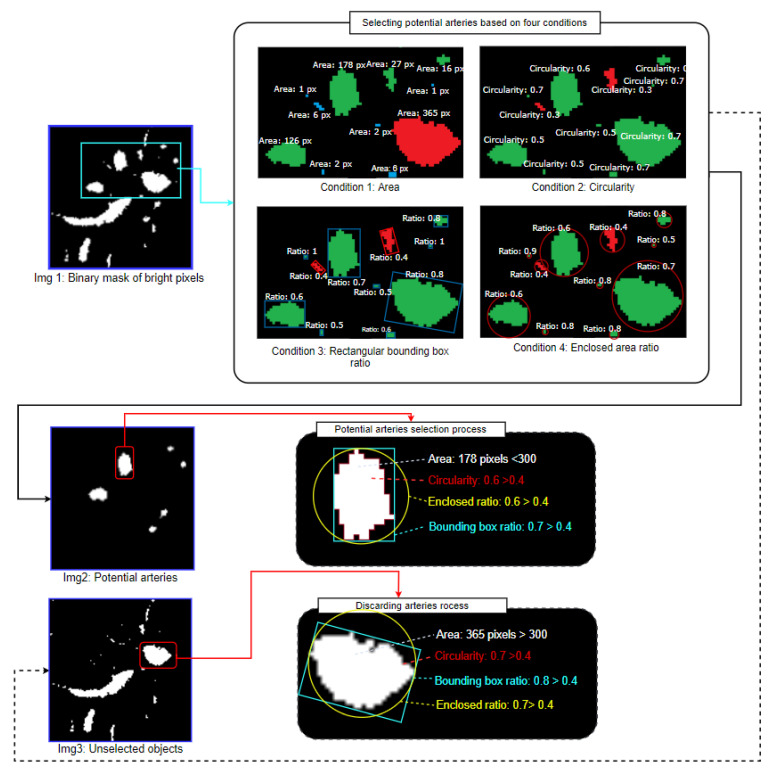
Visual explanation of the four condition-based approaches.

The four conditions are illustrated in Figure 10.

As shown in Figure 10, components having an area <10 are too small and connected components with an area >300 are considered too large (Condition 1). Within this range, the potential arteries can be extracted successfully without losing essential details. Regarding Condition 2, a characteristic of the artery is its approximately circular shape. Therefore, it can be anticipated that objects with a circularity <0.3 cannot be considered potential arteries. For Condition 3, the rectangular bounding box ratio threshold is set to > 0.4. A round object would have a ratio of 1. Finally, for Condition 4, the threshold ratio is > 0.4. The thresholds for each of these conditions are set to such values that any object which might be a potential artery is retained, and these thresholds are obtained through rigorous experimentation. Objects which meet all conditions are selected, and the other objects are discarded. This can be expressed as:

(4) CC=PA: If (C1CC>=10 & C1CC<300) & C2CC>0.3 & C3CC>0.4 & C4CC>0.4

here,

1. *CC* denotes the connected component,
2. *PA* denotes the potential artery,
3. *C*1*CC* refers to the condition-1 applied to the connected component,
4. *C*2*CC* refers to the condition-2 applied to the connected component,
5. *C*3*CC* refers to the condition-3 applied to the connected component and
6. *C*4*CC* refers to condition-4 applied to a connected component.

**Figure 11:**
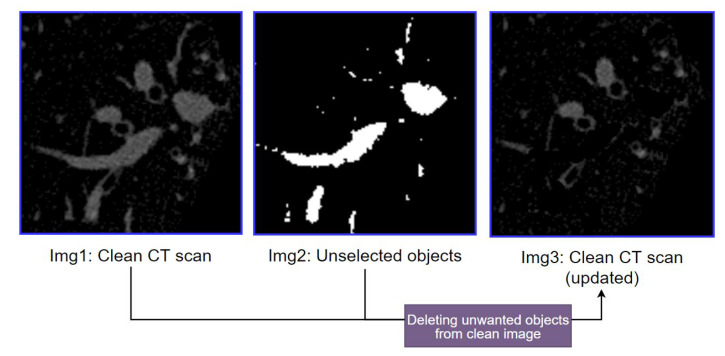
Discarding unwanted bright objects from the clean CT scan.

The output of this process (Img 2 of Figure 10) depicts the selected bright objects considered potential arteries. Furthermore, Img 3 is another output of this process where a mask is generated containing all the discarded bright objects. As these objects do not meet the criteria of Equation (4), these objects are removed from the previously obtained clean CT scan (Img 2 of Figure 8), and the resultant image is considered the updated, clean CT scan (Img 3 of Figure 11).

Figure 11 shows the updated, clean CT scan (Img 3) after discarding unwanted bright objects (Img 2) from the previously achieved clean CT scan (Img 1). This makes finding the BA pairs easier.

### 3.4. Extraction of Objects Adjacent to the Potential Arteries

**Figure 12:**
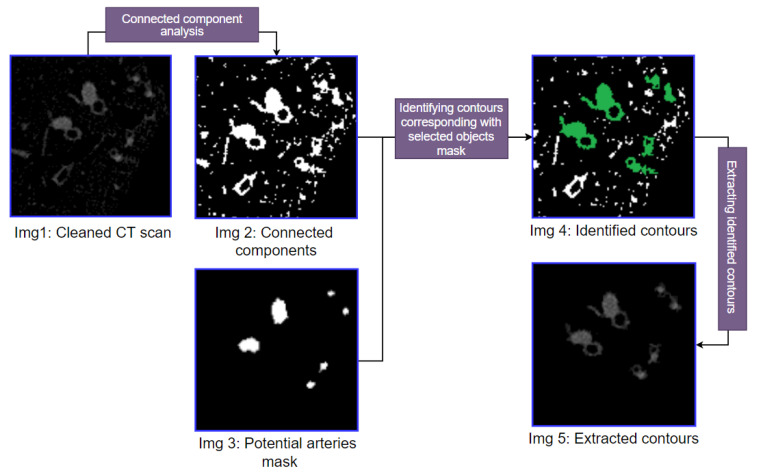
The step-by-step output of extraction of contours of objects accompanied by potential arteries.

In this process, the objects accompanying potential arteries are extracted employing connected component analysis and coordinate matching methods. The connected component analysis technique is applied to the updated, clean image (Img 3 of Figure 11). This results in a binary image containing all the connected components. The coordinates of the potential arteries (Img 3 of Figure 10) are matched with the connected component resultant binary image to extract the contours of the accompanying objects. If a binary mask contour matches the coordinates of a potential artery, it is identified as a potential BA pair, whereas objects not adjacent to arteries remain unidentified. The identified contours are marked in green in Figure 12. These identified contours are extracted from the updated, clean CT image. Figure 12 illustrates this process and shows the step-by-step output.

Figure 12 depicts the output of connected component analysis (Img 2) and the potential arteries (Img 3). In Img 4, all the identified contours are marked in green. These green color marked objects are segmented, and it is found that the BA pairs in the images are successfully extracted. However, other contours, not BA pairs, also remain in the picture, as not all potential arteries will be arteries, or arteries might not be adjacent to bronchi.

### 3.5. Potential Bronchi Extraction

**Figure 13:**
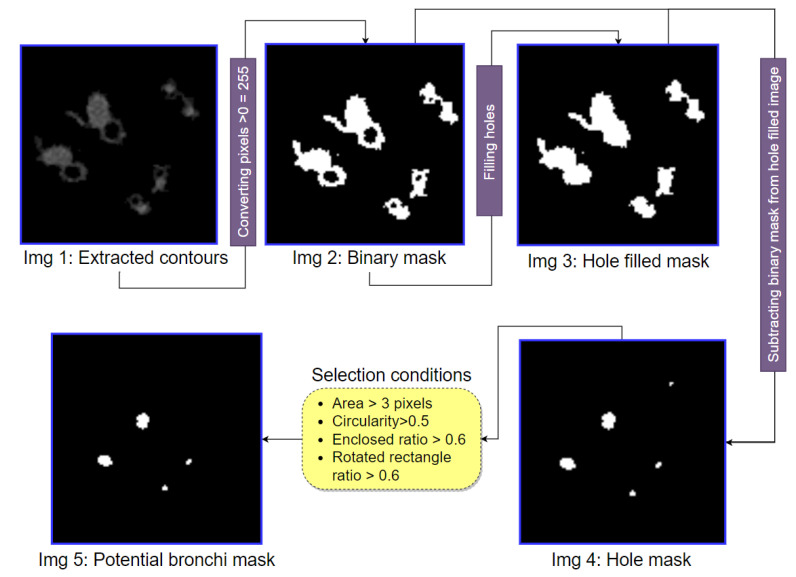
The step-by-step output of potential bronchi detection method.

After extracting the contours of objects accompanying potential arteries, potential bronchi can be identified from the resultant image (Img 5 of Figure 12). In this process, the image is converted to binary, and the hole-filling algorithm is applied to the binary image. The hole-filling algorithm detects all the holes in an image and fills them with the neighboring pixel value [30]. After filling all the holes, a hole-filled binary mask is obtained. The first binary image is subtracted from the hole-filled binary mask resulting in an image in which only the holes (potential bronchi) are visible. Like arteries, one of the main characteristics of bronchi is that they tend to be approximately circular. Hence, the four properties (area, circularity, enclosed circle, rectangular bounding box) previously used to detect potential arteries can also be applied to detect potential bronchi. If a potential bronchus is not circular enough, it will be discarded. Figure 13 illustrates this process with step-by-step outputs.

As can be seen from Figure 13, after applying the hole-filling algorithm to the binary mask of the extracted contours (Img 2), all the holes (potential bronchi) are filled (Img 3). When the first binary mask (Img 2) is subtracted from the hole-filled mask (Img 3), only the holes remain in the resultant image (Img 4). In the hole mask (Img 4), four conditions are applied. After rigorous experimentation, the following thresholds are selected: area >3 pixels, circularity >0.5, enclosed circle ratio >0.6 and rectangular boundary box ratio >0.6. Based on these four conditions, potential bronchi which are close to a circular shape are extracted (Img 5).

### 3.6. BA Pair Extraction

**Figure 14:**
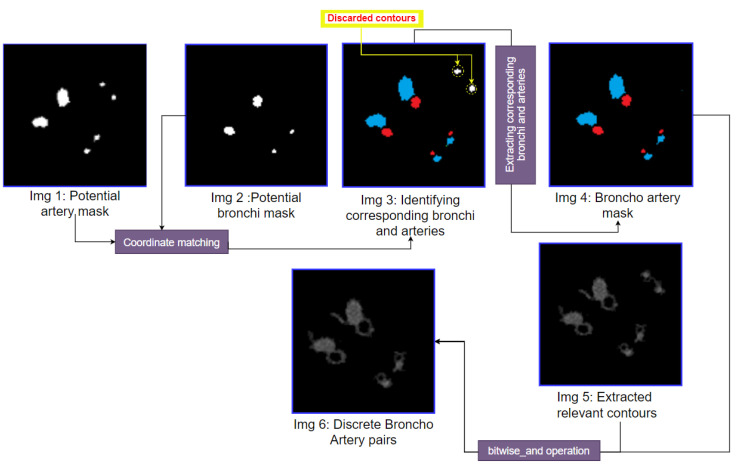
The step-by-step output of the BA pairs segmentation method.

In the previous steps, a potential artery mask (Img 3 of Figure 12) and a potential bronchus mask (Img 5 of Figure 13) are obtained. Combining these two masks, the final bronco-artery pair mask is attained. This bronco-artery mask is merged with the extracted contours image (Img 5 of Figure 12), which results in an output picture containing the discrete bronco-artery pairs. Figure 14 showcases this process with step-by-step outputs.

After matching the potential artery mask coordinates with the potential bronchi mask, the BA pairs can be identified, see Figure 14. In the output image, the arteries are marked in blue, and the bronchi are marked in red (Img 3). Some contours are discarded as these contours do not match the coordinates of potential bronchi and arteries. After discarding these contours, the bronco-artery mask is obtained. Utilizing this mask, the BA pairs are extracted from the extracted contours image (Img 1 of Figure 14). The output image (Img 6) depicts the successfully extracted discrete bronco-artery pairs.

## 4. Analysis of Results

The previously mentioned proposed BA pair segmentation approach is applied to HRCT lung scans of nine patients. The number of detected BA pairs is shown in Table 2.

**Figure 15:**
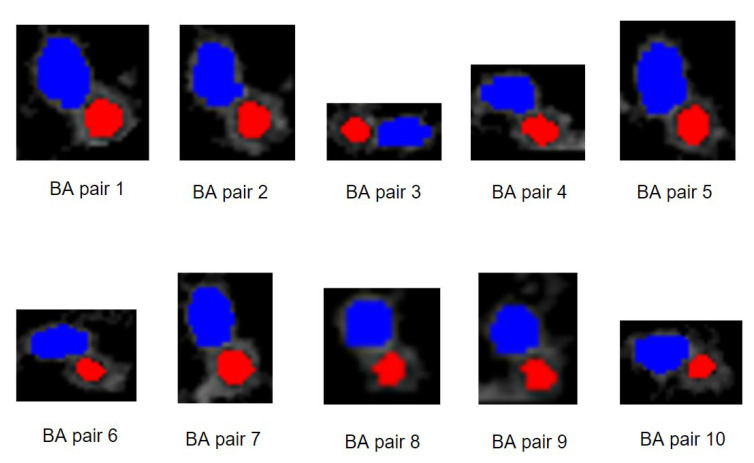
Examples of BA pairs.

Table 2 shows the number of HRCT slices for each patient and the number of detected BA pairs in the right and left lungs. The number of detected BA pairs in the right lung is larger than in the left for all patients. Most BA pairs are detected in the lower areas of the lungs. The largest number of BA pairs is detected for patient 1, with 42 BA pairs in the right lung and 16 in the left lung. For patients 2, 3 and 5, 20–25 BA pairs are detected in the right lung and 10–15 in the left lung. In patient 5, fewer BA pairs (15 on the right, 9 on the left lung) are recorded compared to the previous patients. Regarding the last four patients, 6, 7, 8 and 9, 8–13 BA pairs are recorded across both lungs as fewer slices are available. To visualize the detected BA pairs, ten randomly selected BA pairs identified from various HRCT slices of patient 1 are shown in Figure 15.

Figure 15 depicts BA pairs. Bronchi are marked with a red color, and arteries are marked with a blue color. The locations of these BA pairs are presented in Table 3.

Table 3 contains the slice numbers of the BA pairs of Figure 15 and their coordinates. It can be seen that some BA pairs have similar coordinates as they are located in consecutive slices. For example, BA pair 1 and 2 are located in the right lung region and have similar coordinates in two consecutive slices. On the other hand, BA pair 4 has very different coordinates, as it is located in the left lung. The results of detecting BA pairs for all the subjects are verified by expert radiologists.

**Figure 16:**
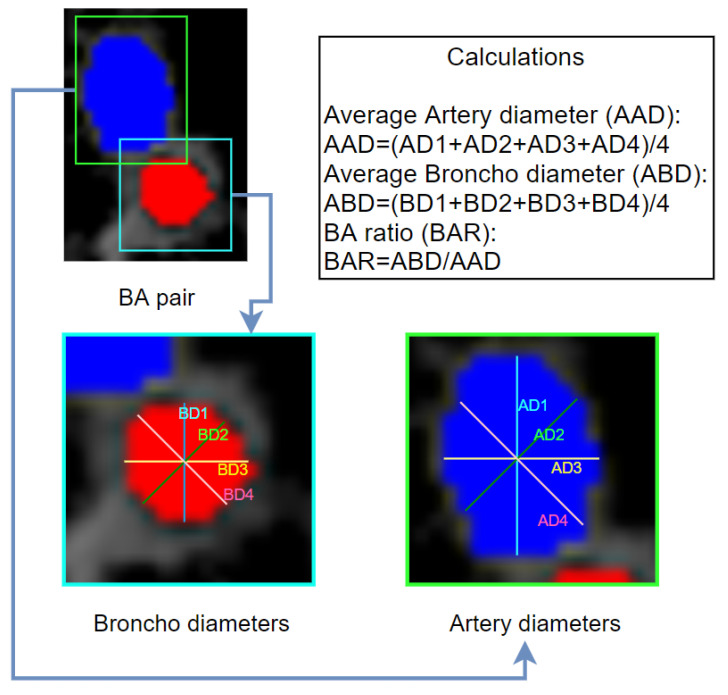
Process of measuring multiple diameters and deriving BA ratio.

The diameters of the bronchi and arteries are measured in an automated manner to determine the BA ratio. When a bronchus becomes inflamed, its size can be considerably greater than its accompanying artery, and together they look like a signet ring on a CT scan. In normal lungs, the size of the artery is the same or slightly larger than its associated bronchus. As the bronchi and arteries are not perfectly circular, four diameters are measured and averaged through an automated process. For the bronchi, the inner diameter (within the bronchial wall) is used. The diameter measurement process is shown in Figure 16.

The center point of the bronchi and arteries is determined to measure the diameters, and four diameters through that center point with angles of 45 degrees between them are taken for averaging. As shown in Figure 16, for the bronchi, a total of four diameters, denoted as BD1 (broncho diameter 1), BD2, BD3 and BD4, are measured. Similarly, four diameters are measured for the arteries, denoted as AD1 (artery diameter 1), AD2, AD3 and AD4. The average bronchus diameter (ABD) and average artery diameter (AAD) can then be calculated. All diameters in this research are measured in pixels (px). Utilizing Equation (5), the BA diameter ratio (*BADR*) is calculated from *AAD* and *ABD*:

(5) $$BADR=\frac{ABD}{AAD}\;\;\;$$

In addition to the diameters, the area of the bronchi (*BAr*) and arteries (*AAr*) is also determined, and the BA area ratio (*BAAR*) is calculated. Here, the area refers to the number of pixels within an object. The formula for calculating the BAAR is presented in Equation (6).

(6) $$BAAR=\frac{BAr}{AAr}\;$$

**Figure 17:**
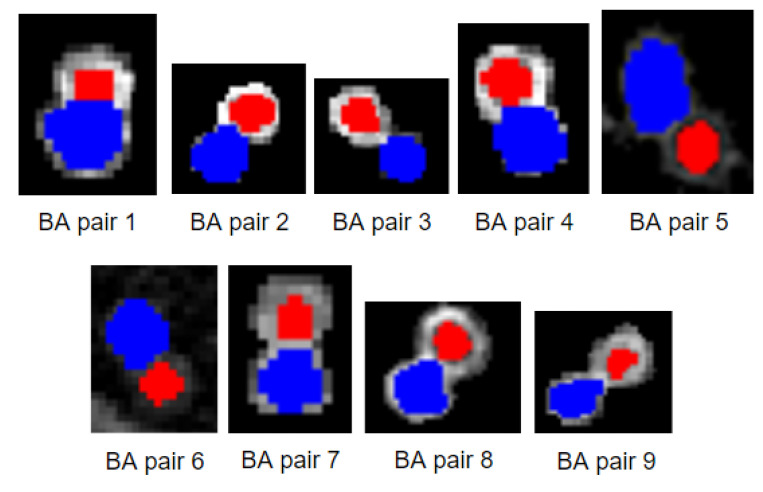
Randomly selected nine BA pairs.

Figure 17 shows nine randomly selected *BA* pairs. Table 4 lists the four diameters for these bronchi and arteries, the average bronchus and artery diameters, the area, and the BA ratio in terms of diameter and area (*BADR* and *BAAR*).

Table 4 shows automated diameter and area measurements of the nine randomly selected BA pairs in Figure 17. It can be observed that the four diameter measurements for individual bronchi and arteries are not equal. The average areas of bronchi and arteries are within the range of 5–42 and 18–125 pixels, respectively. The diameter ratio of the BA pairs falls in the range of 0.51 to 0.70, and the area ratio is 0.20 to 0.63. This way, the BA ratio is derived for each patient, and the average BA ratio is calculated. The average BA ratio is 0.64 and 0.39 for diameter and area, respectively.

To assess the result more rigorously, the BA ratio for the same nine BA pairs is measured and validated by human observers. The measurements were taken in pixels using the medical imaging analysis software ‘Sante Dicom Viewer’ (vendor—Santesoft; location—Nicosia, Cyprus) and each ratio was evaluated. Table 5 depicts the results.

It can be observed from Table 5 that the BA ratios (both area and diamteter) measured using the medical imaging analysis tool ‘Sante Dicom Viewer’ are very close to the BA ratios found with our automated approach. All these nine ratios are confirmed by human observers, which further validates the reliability of the proposed approach.

### 4.1. Performance Validation of the Proposed Approach

To assess the performance of the proposed approach in detecting BA pairs, a deep learning-based classification experiment is conducted. A CT scan dataset is used, and lung disease is classified into three classes (COVID, Non-COVID and CAP). In the study of Azam et al. [31], the significance of BA pairs in diagnosing lung diseases was described, and a classification approach highlighting BA pairs was proposed. They explored four techniques to detect BA pairs and evaluated the results for each technique. It was observed that precise detection of BA pairs resulted in a high classification performance.

**Figure 18:**
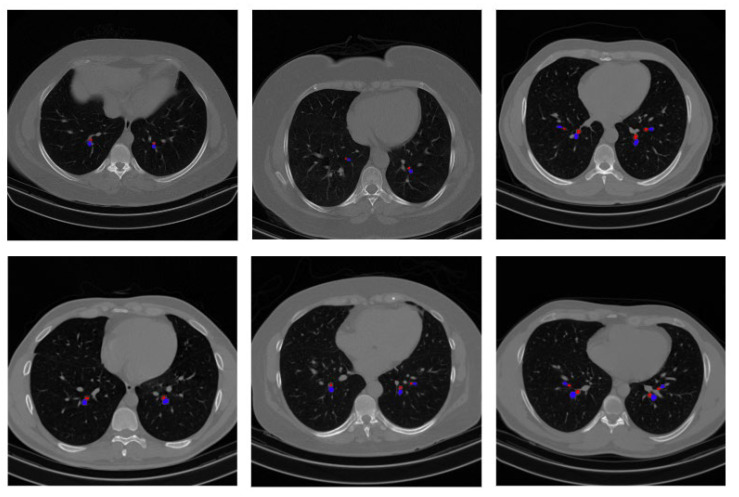
Example output of BA pair detection process on a public dataset.

In this experiment, our proposed approach of detecting BA pairs is applied to the same lung disease CT scan dataset. The output of detected BA pairs on the public dataset is illustrated in Figure 18. Moreover, a total of 100 random scans from the public dataset are validated by the radiologist to confirm that the detected BA pairs are genuine, which further evaluates the effectiveness of the proposed approach.

**Figure 19:**
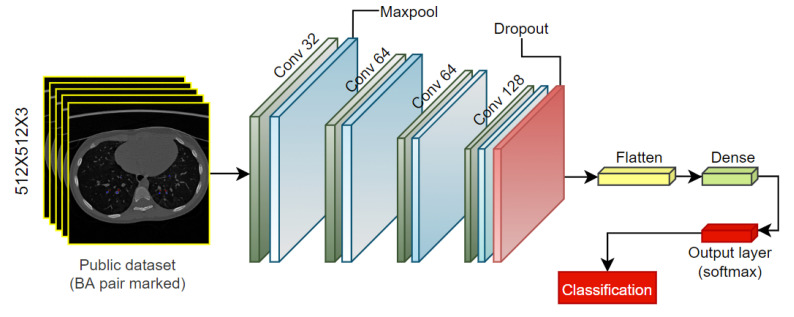
Classification model for a public dataset with highlighted BA pairs.

After the BA pairs are highlighted, the dataset is split into a training, validation, and test set with a ratio of 70:10:20. The classification is carried out using a shallow convolutional neural network (CNN) model (Figure 19). The model has four convolutional layers, each followed by one maxpool layer. The activation function for the convolutional layers is rectified linear unit (ReLU). The kernel sizes for the convolutional and maxpool layers are 3 × 3 and 2 × 2, respectively. The kernels from the first to the fourth convolutional layers are 32, 64, 64 and 128, respectively. After the last maxpool layer, a dropout layer is added with a dropout factor 0.2. Finally, a fully connected layer is added, and the classification is carried out using the SoftMax activation function and the categorical cross-entropy loss function. The model is trained for 200 epochs, with optimizer Adam, a learning rate of 0.001 and a batch size of 32.

Table 6 describes the model’s performance in terms of Training Accuracy, Test Accuracy, Precision, F1 score, Specificity and Sensitivity.

The model has a high classification accuracy of 98.53%, as can be observed from Table 6 while maintaining a 98.59% F1 score. This robust classification performance is achieved as the BA pairs in the CT scans are highlighted, utilizing the proposed BA pair detection method. This validates the robustness of the proposed BA pair detection approach.

### 4.2. Comparison with Existing Competitive Methods

Several studies have been reported on the diagnosis of bronchiactsis, both for children and adults, based on the BA ratio. Table 7 lists the BA ratios found in these studies, along with the age group.

As stated, the exact cutoff of the BA ratio for children and adults is debated. However, several studies suggest that the BA ratio tends to be smaller in children than adults. Table 7 shows the BA ratios for children and adults based on the analysis of existing literature. In the studies of Kapur et al. [9], Thia et al. [32], and Nitin Kapur et al. [33], an assessment of the BA ratio for children was presented. Kapur et al. [9], investigated children of 5–214 months and found a low BA ratio, in the range of 0.437–0.739. Thia et al. [32], conducted a study using data from children with an average age of 52.7 weeks and found a BA ratio in the range of 0.67–0.93. Likewise, Nitin Kapur et al. [33], observed an average BA ratio of 0.626 for children of 3–5 years. Chalwadi et al. [34], and Wu et al. [10], looked at young adults and found average BA ratios of 0.49 and 0.42–0.89, respectively. In the study of Reiff et al. [35], patients aged with an average age of 45 years were investigated, and a BA ratio >1 was found. Matsuoka et al. [22] compared patients of three different age ranges and observed that with increasing age, the BA ratio increases as well. Berend et al. [24] and Park et al. [23] investigated middle-aged patients and found average BA ratios of 0.62 and 0.65, respectively. In our study, HRCT scans of 1–8-year-old children are used. We found BA ratios in the range of 0.51–0.78, with a mean BA ratio of 0.64.

## 5. Discussion

To prevent the progression of bronchiectasis in children, early and accurate diagnosis is crucial. An automated computerized system to detect BA pairs can aid clinicians significantly. This research describes an effective approach to detect and segment BA pairs from CT scans of children. The segmentation of BA pairs is based on several image preprocessing techniques, such as morphological operations, thresholding, and histogram analysis. While detecting arteries and bronchi, a combination of four conditions is utilized, each of which quantifies the roundness of the image objects (bright objects or potential arteries and hole-shaped objects or potential bronchi). Every four conditions propose a different approach to defining if an object is close to a round shape. In this regard, one condition may not suffice for all objects. So, all four conditions serve the purpose of crosschecking the objects and properly identifying the potential arteries/bronchi. In this regard, the thresholds for these conditions are derived through extensive experimentations with a substantial number of CT slices. Through the proposed approach, a sufficient number of BA pairs are detected from both lungs, and the number of BA pairs is quite similar across each patient. Despite minor distortions and roundness issues of bronchi and arteries, the proposed approach can effectively identify the pairs. Moreover, in the previous literatures, in most cases, it is found that only the major and minor diameters are considered to measure the BA ratio. As the intensity level of bronchi and arteries are found to be non-uniform and, to some extent, distorted, in this study, four diameters are measured, which might result in a better approximation of determining a more accurate BA ratio. To present a rigorous assessment of BA ratio measurement, along with the diameter, the area of the BA pairs is also considered. This can be validated from Table 4 that the BA ratios based on diameter fall in the range of 0.51–0.65, and the BA ratio (based on area) falls in the range of 0.25–0.42. Looking at the images of BA pairs presented in Table 4 (Figure 17), the bronchi are smaller than their adjacent arteries, validating the result. Overall, the output of this proposed approach has proven to be quite effective in detecting an adequate number of BA pairs and properly measuring their BA ratio.

In addition, the quantitative outcome of our proposed approach can be integrated into a RIS-PACS platform so that medical specialists can study the information related to BA pairs, including the number of BA pairs, their location, the BA ratio for each patient, and the CT scan. In this way, our analysis can be useful for better-investigating bronchiectasis using HRCT scans. In healthcare systems, PACS is used to store, manage, and share medical images in a secure manner. A radiology information system (RIS) is the electronic health record (EHR) used in radiology, allowing specialists to store and operate data and allocate radiologists’ reports. The RIS-PACS platforms can ensure the security of data sharing with increasing the reliability, precision, and accessibility of information. In this regard, the BA pairs and BA ratio extracted from the patients’ data can be stored on the RIS-PACS platforms, along with each patient’s medical record and HRCT scan. While retrieving a patient’s data from a RIS-PACS platform, the radiologist can consider all information. For instance, radiologists can first look at the patient’s medical information and the original HRCT scan. Afterwards, they can investigate our reports, including the number of BA pairs, their location, and the BA ratios. In this way, time and effort can be saved, and a likelihood of a more accurate diagnosis can be expected. Moreover, if the patient is not diagnosed with bronchiectasis, the data can be shared with other multidisciplinary patient management teams to further evaluate other diseases. Along with medical images, the experts can investigate diseases using our quantitative result of BA pairs.

## 6. Conclusions

This study aims to identify and segment potential BA pairs from HRCT scans of children through an automated approach. The major steps are lung segmentation, image cleaning, potential artery detection, potential bronchi detection and BA pairs extraction. To implement these steps, a suite of image preprocessing techniques such as image denoising, binarizing, largest contour extraction, hole-filling, histogram analysis-based thresholding, balanced histogram thresholding, morphological opening, condition-based thresholding methods, connected component analysis and coordinate matching operations are carried out. When applying this approach, 8–50 BA pairs can be successfully detected across both lungs per patient. The performance of the BA pair detection approach is evaluated through a public CT scan dataset and a deep learning model where a classification accuracy of 98.53% is achieved. The BA ratio is derived for some BA pairs regarding diameters and area.

We aim to detect BA pairs from many patients in future studies to build a large dataset. A deep learning model can be built using this dataset to detect more BA pairs per patient. The inclusion of an adult COPD dataset will be considered in future studies further to increase the number of training and test subjects and improve the identification process of BA pairs. Afterwards, a filtering process will be proposed to identify the distinct BA pairs (BA pairs having only one broncho accompanied by one artery) based on various features such as object area, circularity, and perimeter. After verifying all the detected BA pairs by authorized medical experts, different severity scores can be determined based on the BA ratio, employing deep learning or machine learning techniques such as the decision tree mechanism, which will help the medical experts diagnose bronchiectasis by reducing efforts, time, and error.

## Figures and Tables

**Table 1:** Dataset description of the private HRCT dataset used in this study.

Data Acquisition Information	Technical Parameters Details		Patient Details			
Scanner: Philips Ingenuity Core 64	Parameters	Parameter Value	No.	Sex	Age	Slice/Frames
	Acquisition Mode	Spiral	1	Male	5 years 6 months	429
	Single Collimation Width	0.625 mm	2	Female	3 years 3 months	465
Scanner: Toshiba Aquilion	Total Collimation Width	64 × 0.625 = 40 mm	3	Male	2 years 8 months	553
	Spiral Pitch Factor	1.725	4	Male	1 year 10 months	465
	Kilovoltage Peak	80 kVp	5	Female	9 years 9 months	689
Location: Royal Darwin Hospital, Northern Territory, Australia	Gantry Tilt	0	6	Male	1 year 6 months	54
	Reconstructed Slice Thickness	0.67 mm	7	Male	2 years 6 months	20
	DFOV (Average)	170 mm	8	Female	8 years 4 months	26
	Estimated Dose Saving (Average)	−10	9	Male	1 years 2 months	24

**Table 2:** Results of the proposed segmentation approach.

Patient Number	Total Number of Slices	Number of Detected BA Pairs	
		Right Lung	Left Lung
1	429	42	16
2	465	23	14
3	553	21	11
4	465	25	10
5	689	15	9
6	54	6	2
7	20	6	4
8	26	6	4
9	24	8	5

**Table 3:** Description of example BA patches of patient 1.

BA Pair No.	Slice No.	Co-Ordinate (x, y Position)	Lung Side	Validated by Radiologists?
1	281	179, 259	Right	YES
2	282	177, 258	Right	YES
3	282	163, 279	Left	YES
4	284	357, 234	Right	YES
5	288	176, 254	Right	YES
6	288	156, 276	Right	YES
7	296	174, 250	Right	YES
8	316	175, 235	Right	YES
9	322	174, 232	Right	YES
10	322	149, 265	Right	YES

**Table 4:** BA ratio calculation from both diameter and area measurements.

BA Pair	BD1 (px)	BD2 (px)	BD3 (px)	BD4 (px)	ABD (px)	AD1 (px)	AD2 (px)	AD3 (px)	AD4 (px)	AAD (px)	BADR	BAr (px)	AAr (px)	BAAR
1	4.12	3.00	6.01	6.01	4.79	9.16	8.17	10.00	10.17	9.38	0.51	12.00	61.00	0.20
2	7.24	6.07	7.16	7.03	6.87	9.11	8.21	11.02	9.20	9.38	0.73	33.00	63.50	0.52
3	5.17	5.27	6.04	7.07	5.89	7.03	7.05	8.01	8.06	7.54	0.78	26.00	41.50	0.63
4	7.24	6.16	8.18	8.21	7.45	10.06	9.20	11.03	12.01	10.57	0.70	32.50	76.00	0.43
5	7.08	7.04	9.12	8.12	7.84	10.01	16.00	13.01	14.11	13.29	0.59	42.00	125.00	0.34
6	5.06	5.09	5.20	5.20	5.14	8.04	9.00	8.03	8.04	8.28	0.62	16.00	51.50	0.31
7	2.48	3.00	3.53	4.15	3.29	5.09	5.14	6.12	5.05	5.35	0.62	5.00	18.00	0.28
8	6.22	7.04	7.00	7.13	6.85	9.03	10.00	13.01	13.05	11.27	0.61	35.00	82.00	0.43
9	5.03	5.04	6.25	4.35	5.17	8.03	7.04	11.07	9.14	8.82	0.59	18.00	49.50	0.36

**Table 5:** BA pairs from the automated approach and measured by human observers.

BA Pair No.	BADR: Proposed Approach	BADR: Human Observer	BAAR: Proposed Approach	BAAR: Human Observer
1	0.51	0.53	0.20	0.22
2	0.73	0.70	0.52	0.49
3	0.78	0.82	0.63	0.67
4	0.70	0.65	0.43	0.39
5	0.59	0.60	0.34	0.34
6	0.62	0.62	0.31	0.34
7	0.62	0.59	0.28	0.26
8	0.61	0.57	0.43	0.40
9	0.59	0.58	0.36	0.33

**Table 6:** Performance evaluation of the model.

Performance Matrices	Result (%)	Performance Matrices	Result (%)
Training Accuracy	98.82	F1 Score	98.59
Test Accuracy	98.53	Precision	98.62
Sensitivity	98.45	Specificity	97.74

**Table 7:** Comparison of the proposed approach with various studies stated in the literature review.

No.	Paper	Age Group	BA Ratio
1	Kapur et al. [9]	5–214 months	0.437–0.739
2	Thia et al. [32]	52.7 weeks	0.67–0.93
3	Nitin Kapur et al. [33]	3–5 years	0.626 (average)
4	Chalwadi et al. [34]	0–18 years	0.49 (average)
5	Wu et al. [10]	0–19 years	0.42–0.89
6	Reiff et al. [35]	45 years (average)	>1
7	Matsuoka et al. [22]	21–40 years; 41–64 years; ≥65 years	0.524–0.706; 0.599–0.851; 0.689–0.943
8	Berend et al. [24]	16–60 years	0.62 ± 0.02
9	Park et al. [23]	26–63 years	0.65 (average)
10	Proposed algorithm	1–8 years	0.51–0.78

## Data Availability

The public dataset named- Large COVID-19 CT Scan Slice Dataset is available online: https://www.kaggle.com/datasets/maedemaftouni/large-covid19-ct-slice dataset (accessed on 1 June 2023). Private data is unavailable due to ethical restrictions.
